# Amine- and Amino Acid-Based Compounds as Carbonic Anhydrase Activators

**DOI:** 10.3390/molecules26237331

**Published:** 2021-12-02

**Authors:** Andrea Angeli, Emanuela Berrino, Simone Carradori, Claudiu T. Supuran, Marzia Cirri, Fabrizio Carta, Gabriele Costantino

**Affiliations:** 1 NEUROFARBA Department, Pharmaceutical and Nutraceutical Section, University of Florence, via Ugo Schiff 6, 50019 Sesto Fiorentino, FI, Italy; andrea.angeli@unifi.it (A.A.); fabrizio.carta@unifi.it (F.C.); 2 Department of Food and Drug, University of Parma, Parco Area delle Scienze, 27/A, 43124 Parma, Italy; gabriele.costantino@unipr.it; 3 Department of Drug Chemistry and Technologies, Sapienza University of Rome, P.le A. Moro 5, 00185 Rome, Italy; emanuela.berrino@uniroma1.it; 4 Department of Pharmacy, “G. d’Annunzio” University of Chieti-Pescara, via dei Vestini 31, 66100 Chieti, Italy; 5 Department of Chemistry, University of Florence, via Ugo Schiff 6, 50019 Sesto Fiorentino, FI, Italy; marzia.cirri@unifi.it

**Keywords:** carbonic anhydrase, activators, amino acid, amine, isoform selectivity, coral, protozoa, fungi, learning and cognitive impairment, neurodegenerative diseases

## Abstract

After being rather neglected as a research field in the past, carbonic anhydrase activators (CAAs) were undoubtedly demonstrated to be useful in diverse pharmaceutical and industrial applications. They also improved the knowledge of the requirements to selectively interact with a CA isoform over the others and confirmed the catalytic mechanism of this class of compounds. Amino acid and amine derivatives were the most explored in in vitro, in vivo and crystallographic studies as CAAs. Most of them were able to activate human or non-human CA isoforms in the nanomolar range, being proposed as therapeutic and industrial tools. Some isoforms are better activated by amino acids than amines derivatives and the stereochemistry may exert a role. Finally, non-human CAs have been very recently tested for activation studies, paving the way to innovative industrial and environmental applications.

## 1. Introduction

### 1.1. Amino Acids and Biogenic Amines

Amino acids (AAs) do possess significant roles in Medicinal Chemistry either as free drugs or being constitutive elements within more complex structures in natural products or within synthetic compounds [1,2,3]. The main advantage in dealing with amino acids is to acquire readily available building blocks bearing features such as: (i) orthogonal protecting groups conveniently adjustable by means of well-established synthetic methods (i.e., acylation, alkylation among others); (ii) additional moieties usable to meet the synthetic needs and (iii) chiral centers, with major optical differences between the series produced by eukaryotic or prokaryotic organisms. More importantly all such features are packed into low molecular weight compounds with beneficial effects on their handling and treatment. Among the 22 “proteinogenic” amino acids are included those coded and noncoded, the ones obtained from post-translational modifications [1]. The non-proteinogenic amino acids are not included in protein’s primary sequences and they usually are produced as metabolites from bacteria, fungi, plants or marine organisms. In consideration of the unique structural as well as biological features of many non-naturally occurring amino acids, great efforts have been devoted to the development of appropriate synthetic protocols with the aim to readily obtain innovative building blocks [1,2,4].

Decarboxylation of amino acids, amination or transamination of aldehydes and/or ketones are the main transformations which afford Biogenic Amines (BAs) as naturally occurring organic compounds. BAs are largely present in all organisms and are endowed with variegate physio/pathological features which make them of particular interest within the Medicinal Chemistry field [5].

The purpose of this work is to offer an up-to-date comprehensive overview on AAs, BAs and their synthetic analogues which act in vitro as activators of the metalloenzymes Carbonic Anhydrases (CAs; EC 4.2.1.1) considering this topic is acquiring increased attention among the scientific community for biomedical as well as technological applications.

### 1.2. CA Families

The reversible hydration of carbon dioxide is a minimal transformation which assumes paramount importance within our world of carbon-based life, operating in a water-based medium. All forms of life on Earth share the same biochemistry, which relies on the infinite number of chemical transformations on the “carbon” element. The conversion of carbon into its biologically fruitful form, as well as its detraction, is chemically represented in Equation (1):

CO_2_ + H_2_O ↔ HCO_3_^−^ + H^+^ (1)

At the intracellular concentrations of CO_2_, the uncatalyzed k_cat_ values for the hydration and dehydration steps are of 0.15 s^-1^ and 50 s^-1^, respectively, and thus significantly below the threshold for the biochemical transformations in maintaining the evolution of life [6]. In this context are the Carbonic Anhydrase (CA; EC 4.2.1.1) enzymes which are particularly efficient in speeding-up this equilibrium, thus allowing it in covering any biological need. The abundance of genetic families (i.e., eight and non-correlated to each other) and the multitude of isoforms expressed within some of them clearly reflects the remarkable physio/pathological value of such enzymes which are among the most striking examples of convergent evolution in biology [6,7]. According to the genetic branch, CAs are identified as α-, β-, γ-, δ-, ζ-, η-, θ-, and ι-classes. The first class is the most investigated and comprises isozymes expressed into vertebrates (also including the 15 isoforms reported in humans) algae, coral, protozoa, filamentous ascomycetes and bacterial strains. The main differences occurring between the various α-subclasses are mainly related to secondary and tertiary organization of the apoprotein which in turn determines specific physical/chemical features such as compactness, thermal, chemical and physical stability. On the other hand, the catalytic core is conserved among all the α-family and it consists of a Zn(II) ion tetrahedrally coordinated by three histidine residues and a H_2_O/OH^−^ molecule [8].

The β-CAs catalytic cluster consists of two cysteines and a histidine residue that coordinate the metal ion (i.e., Zn or Co) along with an aspartate and arginine dyad which form a pH dependent gate responsible in switching the enzyme between the catalytically active and inactive forms. Monomeric β-CAs present peculiar α/β folding clusters which can be distinguished in subclasses of the plant- and cab-type. In addition, the monomeric units are organized to afford functionally active quaternary structures up to octamers. The distribution of the β-CAs is quite large in plants, yeasts, bacteria, fungi, and invertebrates, whereas is completely missing in vertebrates [9]. Extensive structural and kinetic investigations on both α- and β-CAs allowed to decipher the mechanistic features proper of the two classes. Important differences are on the release of the proton produced from each single catalytic cycle which also determines its rate. Among the α-family a histidine residue (i.e., His64 according to the human CA II numbering) flips between two opposite conformations called “in” and “out” thus expelling the catalytically generated protons in the enzyme outer space by means of the hydrophilic cavity section [6,10]. As for the β-CAs the mechanisms regulating the extrusion of protons is not completely understood despite a large number of experiments were conducted [11].

The γ-class is widely distributed among the Bacteria and Archaea domains, whereas in eukaryotes have been described only in organisms endowed with photosynthetic abilities. Among this class the CA from *Methanosarcina thermophila* (Cam) still remains the best characterized isoform within this family [12]. X-ray crystallographic experiments clearly showed the active enzyme organized as a homotrimer resulting from the packing of three left-handed β-helices and the catalytic core being placed at the interface between each adjacent monomer [13]. The catalytic core accounts for a metal (II) ion (i.e., Fe, Zn or Co) coordinated according to a trigonal bipyramidal geometry by means of a H_2_O/OH^−^ molecule and two histidine residues from a monomer and one histidine from the adjacent monomer. Although not deciphered in detail, the mechanism for the γ-class is assumed to proceed in analogy to the α-class [14].

As for the δ-family no structural data are currently available and the only relevant information is on the catalytic core which resembles to the cluster in α- and γ-CAs (i.e., three histidine residues coordinating the metal ion). Of note is the metal promiscuity of the δ-class which may be both Zn(II) or Co(II) with no particular effects on the kinetics. The distribution of the δ-CAs appears exclusive in marine diatoms and therefore plays a pivotal role in the CO_2_ sequestration process from the atmosphere at global scale [15]. Genetic analysis of the marine diatom *Thalassiosira weissflogii* revealed sequences encoding for a ζ-CA also named CDCA1. Such an enzyme was also found expressed in *T. pseudonana* diatoms and similar genes were also identified in other diatom species [16,17]. CDCA1 is properly referred as a cambialistic enzyme due to its capability to naturally incorporate either a Cd(II) or a Zn(II) ion at its metal center within a coordination cluster formed of two Cys and one His residues. Despite the metal switch marginally influences the enzymatic performances, which is close to the limit of diffusion [18], studies revealed important differences when in vitro kinetic studies were performed being the Cd-CDCA1 completely insensitive to activation compared with the Zn-CDCA1 enzyme [19]. η-CAs represent a quite interesting family as to date has been isolated only in *Plasmodium* spp. and it seems involved in the biosynthesis of pyrimidine scaffolds by providing HCO_3_^−^ as source of carbon units [20,21]. The catalytic core within the η-class is constituted by two histidine and one glutamine residues able to coordinate the Zn(II) ion according to the classical tetrahedral geometry [22,23]. Despite the high sequence homology between the η- and α-classes, crystallographic studies showed that no metal ion is present and in vitro inhibition experiments confirmed that the enzymatic activity was not affected by classical inhibitors [24]. All such data clearly indicated η-class to possess a distinctive mechanism when compared to other CA-families and yet to be defined.

The β-class was firstly reported in 2016 from the diatom *Phaeodactylum tricornutum* (PtCA1) [25]. Structural and biochemical data indicated such a class to possess many structural features proper of the β-family isozymes (i.e., the catalytic cluster and specific structural domains), but it is endowed with esterase activity which is exclusive of the α-class.

The last CA family that was very recently identified is referred as ι-. Such a CA was firstly isolated from the diatom *Thalassiosira pseudonana* and subsequently found in algae, bacteria, and archaea [26,27,28]. From the structural view point the ι-class is unique since primary structure alignments with all CAs known to date do not show any of the amino acid residues necessary for the metal ion to be coordinated. Such an observation was particularly puzzling considering the elevated kinetic parameters for the hydration rection (i.e., ι-CA from *Burkholderia territorii*: k_cat_ of 3.0 × 10^5^ s^–1^ and k_cat_/K_M_ of 3.9 × 10^7^ M^−1^ s^−1^) and the catalytic activity observed when Zn^2+^ (or Mn^2+^ for ι-CA from diatoms) was added to the culture media for enzyme expression [27]. More recently, Hirakawa et al. identified two novel CAs encoded by an eukaryotic microalga and a cyanobacterium (indicated as BnaCA and AspCA, respectively), showing the same consensus sequence typical of ι-CAs [29]. However, the enzymes were found to be catalytically active without the metal ion, and a putative catalytic mechanism was proposed, with the hydroxyl groups of amino acidic residues (i.e., Thr106, Tyr124 and Ser199) involved in the deprotonation of the active site water [29]. It is unclear whether these metal-free CAs belong to a ι-CA subclass or whether all ι-CAs discovered so far do not need a metal cofactor for the catalytic cycle and the zinc or manganese ion required for the activity only has a structural function [7].

### 1.3. Carbonic Anhydrase Activators (CAAs)

The innovative class of CA activators (CAAs) is increasingly gaining consideration within the biomedical and technological fields. Traditionally the approach to further enhance kinetic performances of such enzymes, which are among the most efficient known so far, has been discarded or just received modest attention mainly as CA inhibitors (CAIs) are endowed with immediate applicability and thus resulting in higher appealing. The activation of CAs was independently reported by several research groups working on biogenic amines such as the histamine, amino acids and small peptides [30,31,32,33]. However, no particular efforts were made with the intent to decipher the CAAs enzymatic mechanism until the early ’90, when the general model of action in Equation (2) has been proposed [34] (E: enzyme; A: activator).

EZn^2+^ − OH_2_ + A ↔ [EZn^2+^ − OH_2_ − A] ↔ [EZn^2+^ − HO^−^ − AH^+^] ↔ EZn^2+^ − HO^−^ + AH^+^ (2)
**Enzyme-activator complexes** (2)

**Figure 1:**
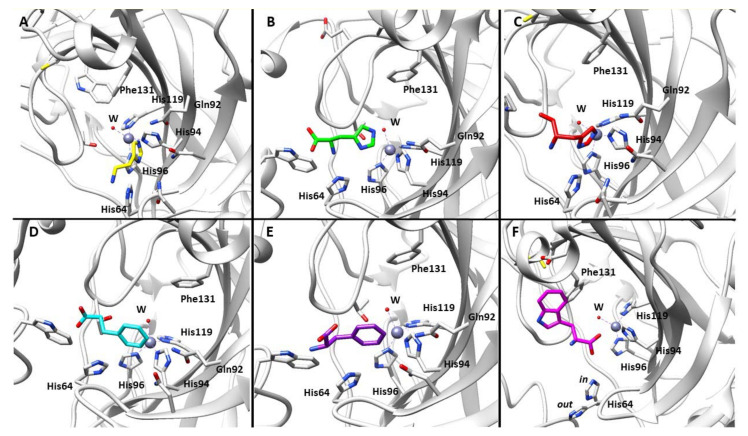
Cartoon views of hCA II/activator complexes as determined by X-ray crystallography. The activators are (**A**) histamine **12** in yellow (PDB 1AVN) [37]; (**B**) D-His **2** in green (PDB 2EZ7) [39]; (**C**) L-His **1** in red (PDB 2ABE) [39]; (**D**) D-Phe **4** in cyan (PDB 2FMZ); (**E**) L-Phe **3** in purple (PDB 2FMG) [42]; and (**F**) D-Trp **6** in magenta (PDB 3EFI) [42].

Such a model stands on the generation of an enzyme-activator complex, which takes part to the rate-determining step of the catalytic cycle. Since the complex is of intramolecular nature, it necessarily means that the proton transfer process is expected to be far more efficient when compared to the same happening via an intermolecular fashion [34,35,36]. The proposed model was also well suited to the pioneering study reported by Tu et al. on the imidazole tail of His64 (assuming the hCA II as model for the α-class) acting as proton shuttle (pKa~7) for the regeneration of the enzymatically active CA species [36]. Among the variegate contributions in support of the proposed mechanism, the most striking ones are represented by the X-ray crystal structures of the hCA II-activator adducts [37,38]. In particular, histamine **12**, the first activator to be crystalized within CA, was found to bind far from the metal ion, in a region also occupied by His64, participating to the complex water network between the zinc ion and the proton-shuttling residue, thus accelerating the regeneration of the enzyme active form. This region, called activator-binding site A, was found to be occupied also by the other activators subsequently crystallized within CA II (Figure 1) [39,40,41], with the only exception represented by D-Trp. This amino acid binds in a distinct region of the active site, called activator-binding site B, although the molecular portions responsible of the proton-shuttling activity (amino and carboxylic groups) are located close to the site A (Figure 1) [42]. Petreni et al. recently performed a very detailed structural analysis of CAAs binding mode, looking at the crystallographic data currently available for the hCA isoform II in adduct with amines and amino acids [43]. In particular, the study aimed to compare the binding mode of CAAs with the one showed by hydrolyzed coumarins, belonging to a non-classic CA inhibitor family. Coumarins, hydrolyzed by the CA esterase activity to 2-hydroxycinnamic acids, occlude the active site entrance and are known to bind in the same region of the enzyme active site occupied by CAAs [44,45]. Both coumarins and CAAs were shown to interact with superimposable amino acid residues, with many water molecules participating to the stabilization of the modulator/enzyme adduct. This phenomenon, unique among all enzymes, highlights the crucial role of specific structural features to be inserted within a CA modulator in the drug design process in order to address the desired biological effect.

**Figure 2:**
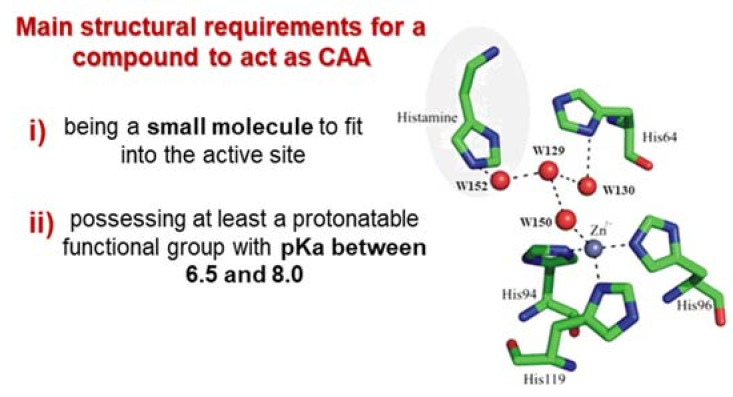
Histamine in complex with hCA II. Hydrogen bonding pathways linking the zinc-bound water molecule, Wat150, to the histamine molecule and to His64 [37].

In particular, a CAA has to be a small molecule fitting into the active site and possessing chemical moieties able to participate to the proton-shuttling process (Figure 2). Such characteristics referred to specific examples will be properly discussed later, because small structural differences may impact a lot on this biological behavior.

## 2. Activation Assay

The in vitro assessment of the CA-activation properties of compounds of interest is properly obtained by measuring the CO_2_ hydration reaction rate means of the stop-flow instrument technique, performed on the method firstly reported by Khalifah [46,47].

The methodology used to perform such an assay relies on monitoring the CO_2_ hydration in Equation (1) within a reaction chamber at a wavelength of 557 nm for a timeframe fixed up to 10 s by using a proper pH indicator (i.e., usually Phenol red). Saturated solutions of the CA substrate (i.e., CO_2_) in H_2_O at 25 °C as well as stock solutions containing the potential CAAs, the enzyme and the indicator are prepared separately using the same aqueous buffer. Usually, CAAs and the recombinant enzyme solutions are preincubated for 15 min at room temperature prior the assay, in order to allow for the formation of the enzyme-activator complex as reported in Equation (1). The activation constant (K_A_) can be obtained by considering the classical Michaelis-Menten equation which has been fitted by non-linear least squares by using PRISM 3 and below reported:

V = V_max_/{1 + K_M_/[S] (1 + [A]_f_/K_A_} (3)

The term [A] refers to the free concentration of the CAA.

Assuming that at the operative experimental conditions the concentration of substrate is lower than K_M_ and that [A]_f_ can be represented in the form of the total concentration of the enzyme ([E]_t_) and activator ([A]_t_), the obtained competitive steady-state equation for determining K_A_ is:

V= V_o_ K_A_/{K_A_ + [A]_t_ − 0.5{([A]_t_ + [E]_t_ + K_A_) − ([A]_t_ + [E]_t_ + K_A_)^2^ − 4[A]_t_ [E]_t_)^1/2^}} (4)

In the equation above, the term V_0_ represents the initial velocity of the enzyme-catalyzed reaction in the absence of activator.

Many advantages are proper of the stop-flow technique applied to the in vitro evaluation of modulators of the CAs and include low cost, easy and direct execution of the experiments, high sensibility as well as data reliability. On the course of the years and with the progression of either technology and information technology the use of such instrumentation as well as data interpretation became easier to access and to validate with beneficial effects on the researchers conducted.

## 3. Activation Studies on Human CAs

### 3.1. Natural and Synthetic Amino Acids and Amines

**Figure 3:**
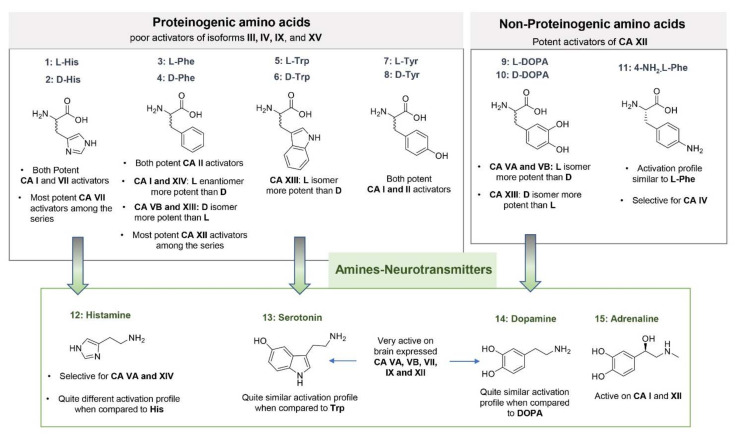
Natural and synthetic (**11**) amino acids and amines **1**–**15** investigated as CAAs.

**Figure 4:**
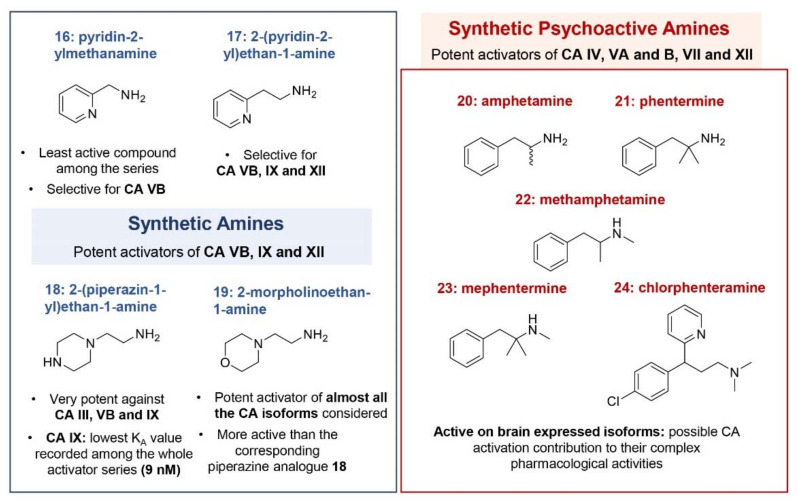
Synthetic amines **16-23** investigated as CAAs.

Biogenic amines and amino acids are by far the most studied CA activators and represent the lead molecules for the design of new compounds endowed of this biological activity [35,38]. The kinetic data (K_A_), collected over the years profiling a pool of 19 amines and amino acids against the 13 catalytically active mammalians CAs (hCA I–XIV and murine (m) CA XV), allow us to draw some general observations about their activating properties and the possible contribution of CA activation to their complex biological activities (Figure 3 and Figure 4) [38]. Five psychoactive substances, including amphetamine and mephentermine, have been also analyzed quite recently against 11 out of the 13 mammalian isoforms, revealing to be able to activate specific CA isoforms, with very low K_A_ values showed by some of them [48]. These results could imply a possible contribution played by CAs in the cognitive effects of such molecules.

As for amino acids, both enantiomeric forms have been studied, revealing some isoforms to be more sensitive to a stereoisomer over the other (high eudysmic ratio). This enantioselective behavior is very interesting and only observed for some of the amino acids considered.

Strong enantioselectivity was shown by Phe, whose L-isomer **3** was largely more active on hCA I than its D-isomer **4** (K_A_ = 0.07 μM and 86 μM, respectively). The latter was found to act as a better activator of CA VB (K_A_ = 0.07 μM) and XIII (K_A_ = 0.05 μM) instead, when compared to the L-analogue **3**. The XIII isoform was better activated also by the D-isomer of Trp **6** (K_A_ = 0.81 μM), and the D-isomer of DOPA, **10** (K_A_ = 0.81 μM), whereas L-DOPA **9** showed higher selectivity for hCA VA and VB (K_A_ = 0.036 and 0.063 μM, respectively). hCA isoforms I, VA and VB and XIII were found therefore to be the most sensitive to the enantiomeric form of the amino acid considered.

Noteworthy, D-Trp showed a strong selectivity for the hCA VA, VB and XIII among the panel of CA considered. Another interesting observation is that, among the amino acids, only L- and D-His showed significant activation properties towards hCA VII. This brain expressed isoform was instead strongly activated by DOPA and neurotransmitters **12**–**15**, which showed also to potently activate mitochondrial isoforms hCA VA and B, and the transmembrane isoform hCA XII. This is a very important information considering the biological role of neurotransmitters.

It is also interesting to observe that, when compared to their amino acidic precursors, some amines preserved the CA activating properties, showing a quite similar activation profile (i.e., Serotonin when compared to Trp), whereas for others (i.e., Histamine when compared to His) the activating properties against the CA isoforms considered were not retained. This phenomenon can be explained considering that for some amino acids (i.e., L-/D-His) the carboxylic group plays an important role in reinforcing the ligand binding to the target, as shown by the available co-crystallographic structures within hCA II [37,39,43]. In terms of biological meaning, these data could also contribute to confirm the different role and activities played by these endogenous molecules, probably mediated by different CA isoforms.

Synthetic amines **16**–**19** and psychoactive substances **20**–**24** both shared with neurotransmitters the poor activity against the cytosolic isoforms hCA I and II, and the low K_A_ values against hCA VA and B. Specifically, synthetic amines **16**–**19** revealed to be very potent activators of hCA IX (K_A_ values ranging from 9 nM to 1.07 μM), an isoform not (or very poorly) activated by all the other compounds considered so far. Morpholine compound **19**, in particular, registered the lowest K_A_ value among the series (9 nM). As for psychoactive substances **20**–**24**, stronger activity was observed against hCA IV, VA and VB, and CA VII, with K_A_ values ranging in the high/medium nanomolar range [48]. Activation of brain expressed isoforms could contribute to the observed complex pharmacology of these substances. In general, very interesting behavior can be observed for some of the compounds here analyzed against hCAs, with a quite complementary activation profile showed by amines and amino acids. For completeness, a table reporting the K_A_ values obtained for compounds **1**–**24** is here reported (Table 1).

Although the SARs drawn for these compounds highlighted for some of them a preferential inhibition of an isoform over the others, for most of the amines and amino acids considered a quite flat activation profile can be observed. With the aim to enhance the selectivity towards specific CA isoforms and also to generate compounds with no structural relation with autacoids, many efforts have been made in the years by Medicinal Chemists in the field of CA activators, with different synthetic approaches developed so far.

### 3.2. Synthetic Manipulations on Amines and Amino Acids

**Figure 5:**
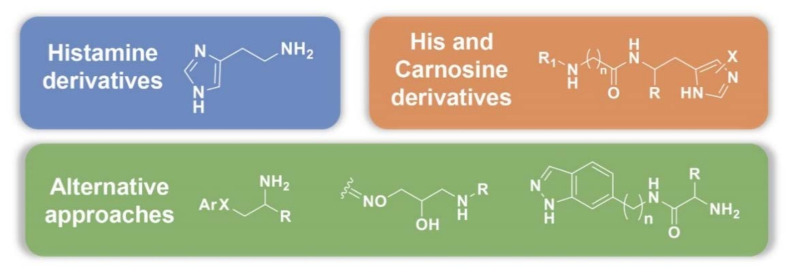
Main design strategies identified so far in the seek for potent and selective CAAs based on amines and amino acids.

Three main design strategies can be found looking at the available literature data (Figure 5). The most exploited drug design approach is represented by the synthesis of histamine analogues, which also included the synthesis of histamine inspired compounds. Another quite used lead is represented by the amino acid His and its β-alanine dipeptide derivative carnosine, along with other His-containing peptides. In the third group we included the compounds obtained applying different approaches, not strictly related to the parent natural amines/amino acids.

**Figure 6:**
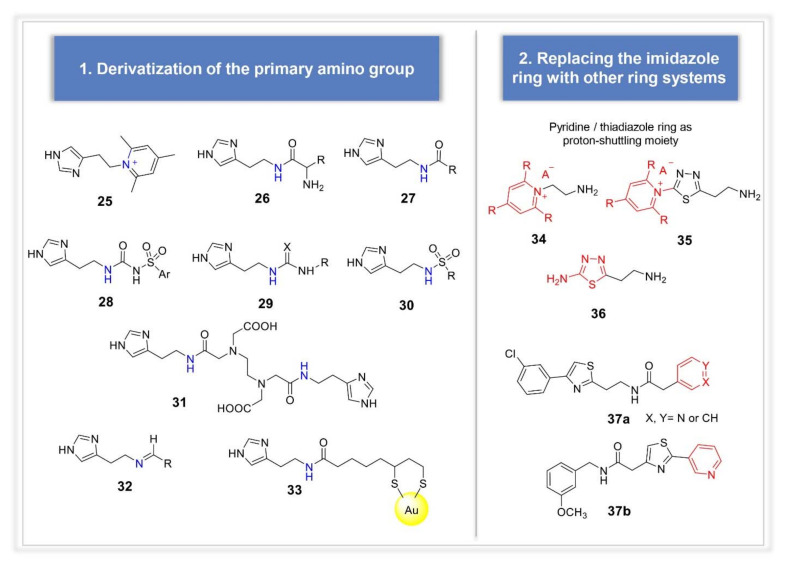
Synthetic analogues of histamine.

Derivatization of the primary group of histamine can be listed among the first strategies reported in the literature for the design of histamine analogues (Figure 6). Crystallographic studies showed this function to point towards the exit of the cavity, thus not involved in the hydrogen bonding network responsible for the enzyme activation [37]. Carboxamides, triazoles, ureas or thioureas, sulfonamides, arysulfonylureido moieties, acylhistamines and bis-histamines incorporating EDTA moiety were all reported to possess a better activation profile against the isoforms I, II and IV when compared to histamine (compounds **25**–**31**) [49,50,51,52,53]. Histamine Schiff bases incorporating aromatic, heterocyclic, or aliphatic moieties were also reported (e.g., compound **32**) [54], as well as histamine pyridinium derivatives (e.g., compound **25**), although their binding mode within hCA II showed to be very different from that usually observed for activators, even resembling the one of an inhibitor [55]. Primary amino group of histamine was also coupled with lipoic acid to be conjugated with gold nanoparticles (compound **33**), resulting in a very strong CAs activation both in vitro and ex-vivo, in normal blood red cells [56]. Another early explored approach consisted in the replacement of imidazole ring of histamine with other ring systems, such as substituted pyridinium ring, 1,3,4-thiadiazole or a combination of the two (compounds **34**–**36**, respectively) [57]. More recently, this strategy was revived by Rami et al., who designed (hetero)aryl substituted thiazol-2,4-yl derivatives incorporating pyridine as proton shuttling moiety (compounds **37a** and **b**) [58]. Very interesting results were obtained, with particular meaning for compound **37b**, which showed to be very selective for CA VII, over CA I and II.

**Figure 7:**
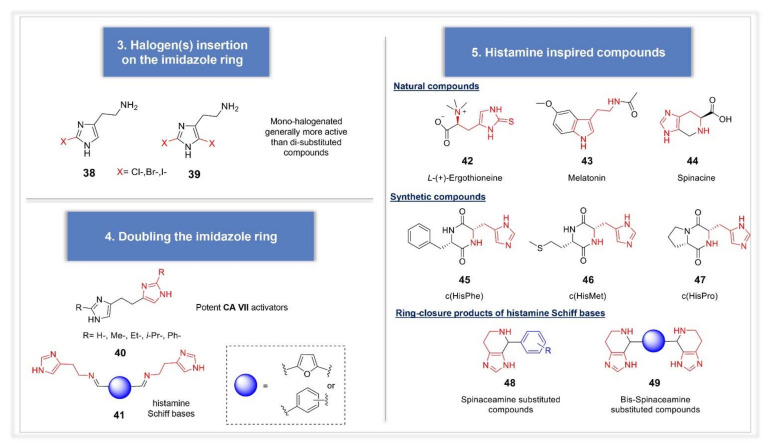
Synthetic analogues of histamine.

Another successful approach was represented by halogen(s) insertion on the imidazole ring, exploiting the withdrawing properties of halogens to affect compounds protonation state and the subsequent interaction with CA binding site (compounds **38** and **39, Figure 7**) [59]. In particular, mono-halogenation gave better results than di-halogenation. Insertion of a second imidazole ring was also explored by Draghici et al., who designed bis-imidazoles of the type **40** in which the two imidazole rings were C-linked via an ethyl linker, bearing substituents of increased steric hindrance in the 2-position of the ring [60] (Figure 7). One imidazole moiety was inserted to work as proton-shuttle, whereas the other as anchoring point to CA active site rim. Small substituents placed in position 2 (H, CH_3_) led to very potent and selective hCA VA and VII activators, suggesting a different binding location of the imidazole ring depending on the isoform considered, driving the observed activation profile. Imidazole ring was found indeed to possess multiple binding sites within hCA II [61]. Following the same strategy, Akocak et al. also reported bis-histamine Schiff bases compounds, bearing different spacers between the two portions. Highly selective hCA VII derivative was represented by the furyl-containing compound **41** [62]. In the same work, the authors also explored the activating properties of bis-spinaceamine derivatives, representing the ring closure products of bis-histamine Schiff bases (compounds of the type **49**). A series of diverse substituted spinaceamine derivatives, which can be considered as an “histamine-inspired compound”, was previously reported by the same authors and the compounds profiled as CA activators, showing nanomolar potencies against hCA VII [63]. Analogously, bis-spinaceamine derivatives showed high potency and selectivity against this isoform, with no significant differences observed among the reported compounds, bearing different spacers between the two active portions. Quantum mechanical (QM) calculations were very recently performed on some of these bis-histamine Schiff bases and bis-spinaceamines, indicating that the activator participates to the proton-shuttling process from the zinc-bound water molecule to the medium and that the electrostatic interactions between the activators and hCA VII are the driving force of the enzyme-activator complex formation [64]. L-(+)-Ergothioneine **42**, Melatonin **43** and Spinacine **44** as well as synthetic compounds **45**–**47**, recently studied as CA activators, should be also mentioned among the histamine inspired compounds (Figure 7) [65]. A strong selectivity for the abundantly CNS expressed hCA VII was observed also for these compounds, which showed to be 10 times more potent than the reference compound histamine. The in vitro results were also corroborated by docking studies [65].

Finally, a large library of histamine receptors (H_1_R–H_4_R) agonists, antagonists and histamine derivatives have recently been profiled against five pharmacologically relevant hCA isoforms, expressed in human brain [66]. Very different potencies and selectivity profiles were observed, most of them showing very low K_A_ values against hCA VII. As expected, compounds devoid of the imidazole moiety or, more in general, of a histamine-related scaffold, did not activate at all the analyzed CAs (K_A_ > 100 μM). The hCA activation property observed for some of these clinically used compounds could explain some of the side effects observed for some of them, or at least contribute to a better understanding of their polypharmacology.

**Figure 8:**
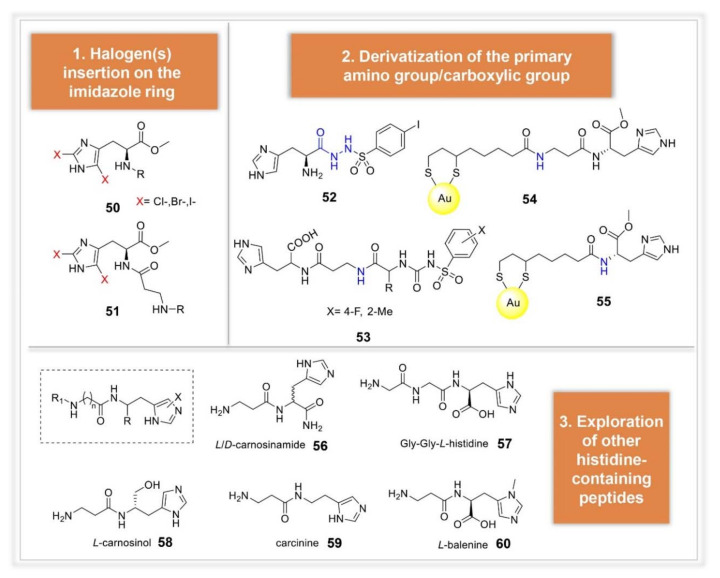
Histidine analogues and histidine containing peptides studied as CAAs.

Another quite well explored lead molecule in the field of CAAs is represented by the amino acid histidine and its β-alanine dipeptide derivative, carnosine. In analogy with histamine, derivatization of the primary amino group of histidine was among the earliest approaches explored. A large set of His and carnosine arylsulfonylureido derivatives was reported in 2002 (compound **53** as representative for the series, Figure 8) [67]. In this study, which provides also the first report of L-carnosine effects on hCAs, very potent derivatives were identified. In particular, compounds incorporating basic amino acids (i.e., Arg or Lys), as well as longer tetrapeptide scaffolds, showed activation constants falling in the nanomolar range. Another synthetic approach explored by Abdo et al. consisted in the synthesis of arylsulfonylhydrazido-L-histidine derivatives, incorporating 4-substituted aryl moieties [68]. This strategy led to compounds less potent than His, with the only exception represented by compound **52**, for which a K_A_ constant of 0.21 μM was recorded against hCA II. Conjugation with lipoic acid to obtain His and Carnosine gold nanoparticles were explored too, as described above for histamine (compounds **53** and **54**, Figure 8) [56]. Halogenation of the imidazole ring of histidine and carnosine was also reported by Saada et al., obtaining compounds of the types **50** and **51**, endowed with different selectivity [69]. Halogenated His derivatives were more potent against hCA I and II, whereas for carnosine this chemical modification improved the activity against hCA VII. A recent study by Vistoli and co-workers, extended the panel of histidine containing peptides to be studied as CAAs, including both natural and synthetic derivatives (compounds **56**–**60**, Figure 8) [70]. The reported compounds were evaluated against hCA I, hCA II, hCA VA and hCA IX, and the obtained data rationalized by docking simulations on hCA II, chosen as model enzyme. The results confirmed the role played by the imidazole ring in affecting the CA activating potencies, while derivatization on the C- and/or N-termini appears to play a more marginal role, mostly affecting the isoform selectivity.

**Figure 9:**
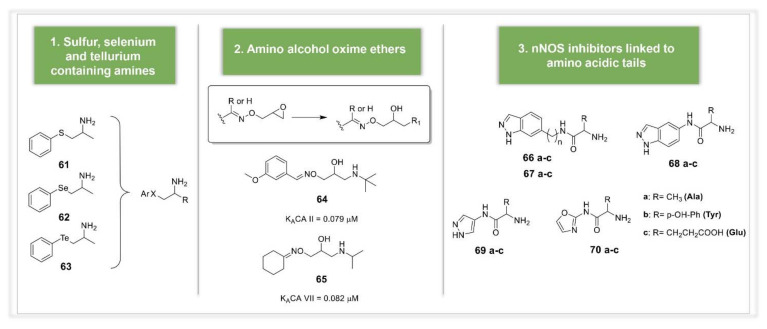
Alternative approaches for the design of CAAs.

Alternative and unconventional approaches for the design on new CAAs were recently reported in the literature by different groups. In 2019, Tanini et al. reported the synthesis and biological evaluation of organic chalcogenides structurally related to the psychoactive drug amphetamine (compounds **61**–**63**, Figure 9) [71]. Compounds were obtained by ring opening of strained aziridine with chalcogen nucleophiles, to give β-arylchalcogeno amines containing sulfur, selenium and tellurium. This quite large series showed good activating properties against the isoforms hCA I, VA and VII and a potent antioxidant activity, especially for selenium and tellurium compounds, which were able to prevent ROS metabolites generation and the consequent cellular stress and damage. These compounds are, therefore, of great interest in the field of neurodegenerative disorders treatment, where the levels of ROS are particular high. More recently, a large series of amino alcohol was reported by Nocentini et al. obtained by ring opening of differently substituted epoxydic oxime ethers with isopropylamine or *tert*-butylamine [72]. These compounds were designed using as lead the β-amino alcohol timolol, previously found to act as CAA [73]. The synthetized compounds were assayed as CAAs against four physiologically relevant hCA isoforms expressed in human brain, showing K_A_ values spanning from the low micromolar to the medium nanomolar range. Compounds **64** and **65** in particular revealed to be highly selective for the isoform CA II and VII, respectively, opening new perspectives in the design of potent and selective CAAs based on the amino alcohol scaffold, as valid alternative to amines and amino acids [72] (Figure 9).

Another novel approach was reported by Maccallini et al., who designed indazole, pyrazole, and oxazole derivatives bearing amino acidic tails, such as alanine (Ala), tyrosine (Tyr), and glutamic acid (Glu) (compounds **66**–**70**, Figure 9) [74]. This strategy was based on the well-known property of heterocyclic compounds and amino acids to activate CAs, coupled to the ability of indazole, pyrazole, and oxazole derivatives to inhibit neuronal nitric oxide synthase (nNOS). These two biological activities could make the designed compounds valuable drug candidates for the treatment of neurodegenerative disorders (i.e., Alzheimer’s disease (AD), Parkinson’s disease (PD)), where an abnormal nitrergic signal was observed, along with a low CA expression, affecting cognition and leading to mental retardation. Among the series, 5-substituted indazole derivative **68b** containing Tyr as CAA moiety showed to be the most promising dual agent, selectively inhibiting nNOS (over iNOS and eNOS) and activating hCA I.

## 4. Therapeutic Applications for Human Health

**Figure 10:**
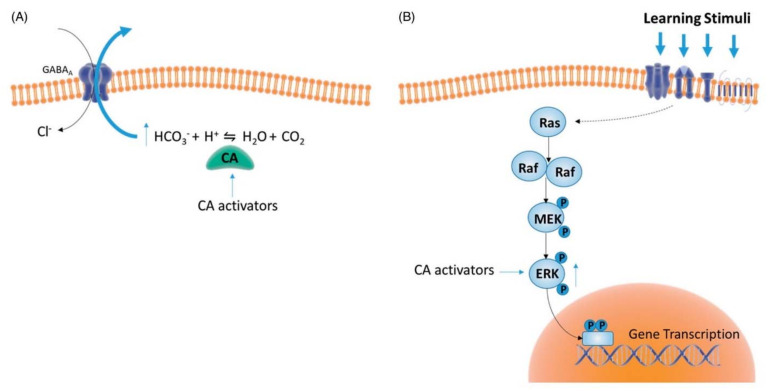
Putative mechanisms underpinning CAs actions on cognition. (**A**) CA activation transforms GABA-mediated inhibition (Cl^−^ conductance) into excitation due to increased HCO_3_^−^ flux through the GABA_A_ receptor channel. Such synaptic transformation allows GABA-releasing interneurons to act as either excitation filters or amplifiers of the neuronal network [76]. (**B**) CA activators increase ERK phosphorylation [78], which in turn regulates the activity of nuclear transcription factors promoting gene transcription, an essential step for consolidation of different learning stimuli [81]. The CA isoforms as well as the cellular mechanisms related to CA-induced modulation of ERK activity were not identified yet. Reproduced from [80].

As above mentioned, CAAs main therapeutic applications for human diseases are in the field of pharmacological enhancement of synaptic efficiency, spatial learning, and memory. Involvement of CAs in cognitive function was already proposed by Sun and Alkon [75,76], whose theories were supported by previous evidence of a significantly diminished activity of CAs in patients affected with AD, when compared to age-matched controls [77]. These studies have been resumed by Blandina’s group, who investigated from a mechanistic point of view CA involvement in cognition [66,78,79,80]. In particular, the importance of CA activity in spatial and fear memory formation was demonstrated by using blood brain barrier (BBB) permeant (AAZ) and impermeant (C18, a pyridinium perchlorate compound) CA inhibitors, and a CAA, D-Phe. Permeant but not impermeant inhibitor, which does not cross the BBB, was able to impair short-term novel object recognition memory and consolidation of fear extinction memory. CA activation using D-phenylalanine led instead to improved performances in memory task, generating a long-term memory that persists up to 24 h after training. CA activation has therefore a crucial role in transforming short-term learning into long-lasting memory, and this effect was related to CA activation-dependent increased pERK expression, as one of the possible CAA underlying mechanisms (Figure 10) [78]. Activation of ERK pathways in the cortex and in the hippocampus triggers the genomic response in neurons and leads to structural synaptic changes that facilitate memory encoding [81]. Analogously, in fear memory extinction models the selective inhibition of CAs in the brain correlates with impairments of extinction whereas activation has beneficial effects.

These studies pave the way for pharmacological application of CAAs in the management of post-traumatic stress disorders (PTSD), phobias and generalized anxiety. Potentially, CAAs could be also considered as future new tools for the treatment of memory associated symptoms from neurodegenerative diseases and aging. Another very interesting application of CAAs is in the field of tissue engineering, mainly explored by Müller’s group [82,83]. Carbonate deposition is animals (i.e., mollusk shells) is known to rely on the CA-driven HCO_3_^−^ formation as rate limiting step. Studies on human osteogenic SaOS-2 cells exposed to Ca(HCO_3_)_2_ in vitro revealed an increase of Ca-deposit formation and upregulation of the *CA II* gene expression. Moreover, addition of a CA inhibitor (AAZ) prevented Ca-deposit formation [83]. Using sponges as a biological starting material, the presence of CA activators led to an enhanced formation of calcium carbonate, which acts as bioseeds for the precipitation of calcium phosphate, in the bone formation process [82]. The discovery that CAs are highly involved in CaCO_3_ deposition in vitro and likely in vivo opened the way to the exploration of CAAs for increasing bone formation.

## 5. Natural and Synthetic Amino Acids and Amines Activating Non-Human CAs

The last review on these biomolecules was elegantly reported by Akocak and Supuran in 2019 [3], but due to the overwhelming published literature, we performed an update including the most recent discoveries in this field. The development of non-human CAAs has been much limited due to their complicated pharmacology and catalytic cycle (e.g., Zn(II) can be substituted by other metal ions or can be catalytically inactive) compared to the design of CAIs (Table 2). Indeed, the extrapolation of SARs is rather complex compared to human enzymes due to the differences in the active site of the isozymes present in a large number of organisms.

**Figure 11:**
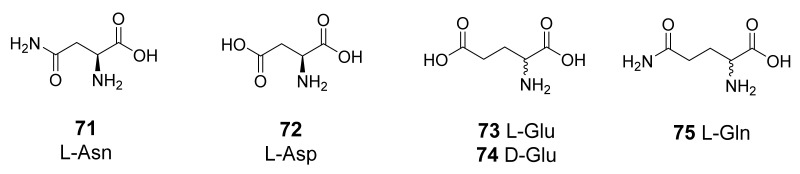
Amino acids **71**-**75** recently introduced to better study the activation mechanism of non-human CAs.

As seen above, the most extensively studied CAAs usually belong to amines and amino acids as chemical scaffolds and compounds **1**–**19** (Figure 1) were always tested for their activation of non-human CA isoforms. Moreover, other amino acids (**71**–**75**, Figure 11) were recently introduced to better understand the general structural requirements for such activity. The use of the same panel of activators by the same research group can be considered a straightforward strategy to rationalize the differences within the series of related targets and to gain more robust information about the impact of the stereochemistry in the biological interaction.

Lastly, tripeptides were also designed to enlarge the chemical space suitable for the activation of these enzymes.

From a synthetic point of view, non-human CAs activators are not obtained by means of sophisticated organic reactions, because their structures are largely derived from natural amino acids. A recent and promising approach could come from dynamic constitutional strategies developed to produce enzyme-dynamic combinatorial systems using simple synthetic building blocks with a high rate of variability and versatility [108]. Collectively, the knowledge of the K_A_ values of these derivatives, not only regarding the human CAs but also considering the non-human isozymes, allowed us to assess the most essential chemical functions to achieve a good selectivity index. Indeed, for comparative purposes, we have also reported K_A_ values obtained with the most ubiquitous α-CAs isoforms (CA I and II) reported at paragraph 3. The criteria for selectivity are important due to the fact that non-human CAs are present in seven out of the eight different genetic families known to date (only θ-CAs identified in marine diatoms are unexplored yet for activators), isolated from pathogenic and non-pathogenic organisms.

The CA activation in these species, albeit not useful for therapeutic use, can improve our understanding of the catalytic mechanism or the involvement of the proton shuttling as in human CAs. In pathogenic species, this information strengthens the functional role of their specific CAs about evolution, survival, metabolism and virulence, providing comprehension of how these factors are influenced by modulators of CA activity to develop new therapeutic strategies avoiding drug resistance that has emerged for most clinically used anti-infectives.

On the other hand, CAs from coral, such as CruCA4, were shown to be involved in the biomineralization process and in vivo enhanced growth rates of coral skeletons [106]. Conversely, in diatoms these CAs seem to be involved in the carbon acquisition pathway leading to a hydrolase activity of different substrates [109]. Finally, the thermostable and very active α-CAs from the thermophilic bacteria *Sulfurihydrogenibium yellowstonense* YO3AOP1 and *Sulfurihydrogenibium azorense* (SazCA) provided industrial applications for biomimetic CO_2_ capture processes [84].

For sake of clarity, we have collected the kinetic data of compounds **1**–**19** to define preliminary SARs clustered in families, keeping into consideration the lack of X-ray crystal structures of CAAs in complex with any CAs other than hCA II (Table 3, Table 4, Table 5, Table 6).

Table 3 collected the activation profiles of five α-CA isoforms belonging to two corals (STPCA, CruCA4), three bacteria (VchCAα, SspCA, SazCA) and one protozoan (TcCA), which all showed very different sensitivity to this panel of amino acids and amines. The comparison of the biological in vitro data of coral CAs STPCA (*Stylophora pystillata*) and CruCA4 (*Corallium rubrum*) provided the general higher sensitivity in the low nanomolar range of CruCA4 to CA activators, especially biogenic amines. Only D-DOPA **10** represented the most interesting STPCA activator (K_A_ = 0.18 µM) within the series and with two orders of magnitude with respect to its enantiomer (**9**, L-DOPA, K_A_ = 15 µM).

Regarding bacterial α-CAs, whereas VchCAα (*Vibrio cholerae*) was poorly activated by this set of molecules, kinetic data referred to SazCA (*Sulfurihydrogenibium azorense*) and SspCA (*Sulfurihydrogenibium yellowstonense*) except for compound **4** (D-Phe, K_A_ = 5.1 µM) showed a potent activation down to K_A_ = 0.002 µM. L-Phe, D-His, L-Trp and D-Trp were endowed with the best values, thus providing useful insights into the possibility to boost this enzyme for industrial and technological biomimetic CO_2_ capture. Finally, TcCA from the pathogenic *Tripanosoma cruzi* was strongly activated by non-proteinogenic amino acids (**9**–**11**) and the morpholine derivative **19**.

The β-CAs family was also largely explored. They are well-represented in the Fungi kingdom as reported in Table 4. Collectively, this set of nitrogen-containing compounds had a limited activating activity, except for the fungal isoform MreCA (*Malassezia restricta*). Non-proteinogenic amines and amine neurotransmitters were preferred to obtain nanomolar activation constants. If we consider other β-CAs from bacteria and protozoa (Table 5), BpsCAβ, VchCAβ (*Vibrio cholerae*), BsuCA (*Brucella suis*) and LdcCA (*Leishmania donovani chagasi*) were the most sensitive to these compounds. Synthetic amines (**16**-**19**) were shown to be potent activators of LdcCA isoform, thus representing useful and selective tools to better understand the role this enzyme has in the complex life cycle of *Leishmania*. FtuCA (*Francisella tularensis*) was the least affected β-CA (K_A_ > 30.5 µM) within the series.

Among γ-CAs included in Table 6, these selected amine derivatives and amino acids displayed a strong preference for VchCAγ (*Vibrio cholerae*) and BpsγCA (*Burkholderia pseudomallei*) with K_A_ values in the low nanomolar range, albeit a general SAR cannot be depicted. More in detail, the γ-class in *V. cholerae* was the most sensitive to these CAAs with respect to the other two genetic families. The rest of γ-CAs were not significantly affected by these CAAs.

The activation data for the other families (δ-, ζ-, and η-CAs) underlined the differences in the tertiary structure of these isozymes leading to a moderate sensitivity to both amines and amino acids. A new entry in this field was the recently discovered ι-CA (*Burkholderia territorii*). Despite most of the CAAs showed rather flat activating efficacy with K_A_ values ranging between 3.9 and 45.6 μM (compounds **1**–**19** and **71**–**75**), the results suggested that small structural changes (e.g., the stereogenic center) in the compounds can induce important modifications of their CA activating properties. Moreover, the activation profile of these bacterial ι-class was very different from those of human CA I and II.

Beyond the well-known CAAs **1**–**19** and **71**–**75**, Stefanucci et al. [87] explored six newly synthesized tripeptides (**76**–**81**) as trifluoroacetic acid salts of general formula NH_2_-Xaa_1_-Xaa_2_-Xaa_3_-NH_2_ (Table 7, being the amino acids of L series). First of all, they were totally inactive against human CA I and II (K_A_ > 50 µM), whereas they displayed an interesting activation profile toward CAs from *V. cholerae* (VchCAβ and VchCAγ), mtCA3 from *M. tuberculosis* and BpsCAγ (from *B. pseudomallei*).

VchCAβ and BpsCAγ were efficiently activated by these tripeptides in the range 0.21–10.1 µM. The most promising derivatives shared a common skeleton at position Xaa_2_ (Asp) and Xaa_3_ (Ser). The other two CA isoforms (mtCA3 and VchCAγ) were less affected by these compounds, which displayed a better preference for *V. cholerae* CA isoforms.

CAAs for these isozymes can be useful for two main reasons. Firstly, taking into account the paramount role in coral reef ecosystems of organisms using CAs in symbiosis or calcification processes, the activation of these isoforms can improve or sustain photosynthesis and biomineralization. These two aspects have gained attention to the emerging concern due to the harmful ocean acidification, climate changes and marine pollution and led the scientists to better understand physiological pathways towards carbon-concentrating mechanisms and how to improve them [107]. Secondly, in a larger effort for green CO_2_-capture, -utilization and -storage, microbial CAs have been engineered to develop more efficient technologies for an improved mineral carbonation. Moreover, this result can be also achieved by the use of activators avoiding low stability under industrial conditions [110].

## 6. Conclusions

The field of carbonic anhydrase activators has been underexplored so far due to the skepticism that scientists experienced regarding an enzyme which usually improves the rate of an important biological reaction. However, after the preliminary demonstration of this phenomenon with a handful of different available hCA-activator X-ray structures, activators were seen not only as tools to unravel the enzymatic mechanism (favoring the proton shuttling), but also as therapeutic and industrial agents. Indeed, CA-deficiency syndromes for many human isoforms (CA I, II, IV, VA, XII and XIV) and the tissue engineering field make these activators rather promising, especially in the memory therapy and to obtain artificial bone fragments, respectively. This approach has been corroborated by in vivo studies of enhancement of spatial memory and learning [66].

Beyond anions and sulfonamides as inhibitors, amino acids and amine derivatives were the most explored compounds and scaffolds to generate rather potent and selective CAAs among the 13 mammalian isoforms and the others belonging to different living organisms. Most of the compounds were derived from proteinogenic and non-proteinogenic amino acids, and neurotransmitters, by the introduction of moieties characterized by specific pK_a_ values and lipophilicity. All the compounds reported in this review were published performing the same procedure and using the same instrument, thus ensuring a strong reproducibility of the data and robust SARs. Further significant developments are expected from this less explored field in medicinal chemistry, especially regarding the θ-CA family and the availability of X-rays analysis of CA-activator complexes with not human isozymes.

## Figures and Tables

**Table 1:** In vitro α-CA activation data (K_A_) with amine derivatives and amino acids (**1**–**24**). Each value is the mean from three different determinations with errors in the range of 5–10% [38].

cmpd	K_A_ (μM)												
	hCA I	hCA II	hCA III	hCA IV	hCA VA	hCA VB	hCA VI	hCA VII	hCA IX	hCA XII	mCA XIII	hCA IV	mCA XV
**1**	0.03	10.9	35.9	7.3	1.34	0.97	32	0.92	9.71	37.5	0.13	0.9	32.1
**2**	0.09	43	1.13	12.3	0.12	4.38	13	0.71	12.5	24.7	0.09	2.37	14.1
**3**	0.07	0.013	34.7	36.3	9.81	10.45	1.23	10.93	16.3	1.38	1.02	0.24	33.4
**4**	86	0.035	15.4	49.3	4.63	0.072	16	9.74	9.3	0.37	0.051	7.21	9.5
**5**	44	27	20.5	37.1	1.13	0.89	-	57.5	37.5	23	16	16.5	13.5
**6**	41	12	19	39.6	1.24	1.35	-	39.6	43.6	28.1	0.81	18	8.7
**7**	0.02	0.011	34.1	25.1	2.45	0.044	-	20.3	25.3	25.8	-	21.8	8.9
**8**	0.04	0.013	-	-	-	-	-	-	-	-	-	-	-
**9**	3.1	11.4	13.5	15.3	0.036	0.063	-	58.3	51.3	1.67	43	12.1	6.5
**10**	4.9	7.8	28.7	34.7	4.59	3.71	-	34.7	54.7	0.89	0.73	36.8	4
**11**	0.24	0.15	43.2	0.079	2.76	2.17	-	18.7	48.7	1.09	-	2.9	16.3
**12**	2.1	125	36.9	25.3	0.01	3.52	6.5	37.5	35.1	27.9	4.6	0.01	18.5
**13**	45	50	0.78	3.14	6.33	0.11	-	0.93	33.1	0.3	0.51	6.5	7.5
**14**	13.5	9.2	33.2	30.9	0.13	7.85	21.1	0.89	0.92	0.67	27	14.6	7.1
**15**	0.09	96	36.4	45	-	-	-	-	60	0.87	-	36.1	6.9
**16**	26	34	1.03	5.19	23.56	0.24	-	43.7	1.07	41.5	3.8	21.7	11.6
**17**	13	15	1.1	7.13	7.62	0.094	-	27.8	0.013	0.69	46	6.9	11.9
**18**	7.4	2.3	0.32	24.9	6.04	0.91	9.54	32.5	0.009	48.3	54	18.3	10.4
**19**	0.14	0.19	0.091	1.3	0.089	1.15	42	64.3	0.43	0.24	0.013	5.4	9.3
**20**	>150	>150	-	0.094	0.81	2.56	>150	0.91	>150	0.64	24.1	9.15	-
**21**	>150	>150	-	0.074	0.53	0.62	>150	0.89	34.6	3.24	54.2	12.7	-
**22**	>150	>150	-	0.051	0.92	0.78	>150	0.93	>150	0.8	25.6	7.38	-
**23**	>150	>150	-	1.03	0.37	0.24	>150	0.64	25.8	6.12	48.3	18.1	-
**24**	>150	>150	-	0.055	0.31	0.75	>150	0.098	34.1	0.97	79.5	6.81	-

-: not determined; cmpd: compounds.

**Table 2:** Non-human species characterized by different CA families and tested for the activation mechanism.

Species	CA Family (Acronym)	Ref.
**Gram-Positive and Gram-Negative Bacteria**		
*Sulfurihydrogenibium yellowstonense*	α (SSpCA)	[84]
*Sulfurihydrogenibium azorense*	α (SazCA)	
*Vibrio cholerae*	α (VchCAα), β (VchCAβ), and γ (VchCAγ)	[85,86,87]
*Mycobacterium tuberculosis*	β (mtCA3 or Rv3273 CA)	[87,88]
*Methanobacterium thermoautotrophicum*	β (Cab)	[89]
*Escherichia coli*	β (EcoCAβ)	[90]
*Brucella suis*	β (BsuCA1)	[91]
*Francisella tularensis*	β (FtuCA)	[91]
*Burkholderia pseudomallei*	β (BpsCAβ)	
*Methanosarcina thermophila*	γ (Cam)	[89]
*Burkholderia pseudomallei*	γ (BpsγCA)	[87,92]
*Pseudoalteromonas haloplanktis*	γ (PhaCA)	[93]
*Colwellia psychrerythraea*	γ (CpsCA)	[93]
*Burkholderia territorii*	ι (BteCAi)	[94]
**Fungi and Yeasts**		
*Saccharomyces cerevisiae*	β (scCA)	[95]
*Candida albicans*	β (CaNce103)	[96]
*Cryptococcus neoformans*	β (Can2)	[96]
*Candida glabrata*	β (CgCA)	[97]
*Malassezia globosa*	β (MgCA)	[98]
*Malassezia restricta*	β (MreCA)	[99]
**Protozoa**		
*Trypanosoma cruzi*	α (TcCA)	[100]
*Leishmania donovani chagasi*	β (LdcCA)	[101]
*Entamoeba histolytica*	β (EhiCA)	[102]
*Trichomonas vaginalis*	β (TvaCA1)	[103]
*Plasmodium falciparum*	η (PfACA)	[104]
**Microalgae**		
*Thalassiosira weissflogii*	δ (TweCAδ) and ζ (TweCAζ)	[19,105]
**Corals**		
*Corallium rubrum*	α (CruCA4)	[106]
*Stylophora pystillata*	α (STPCA)	[107]

**Table 3:** In vitro α-CA activation data (K_A_) with a panel of amine derivatives and amino acids (**1**–**19**). Each value is the mean from three different determinations with errors in the range of 5–10%.

Compound	K_A_ (μM)					
	STPCA	CruCA4	VchCAα	SspCA	TcCA	SazCA
**1**	28.0	36.9	43.2	0.11	11.3	0.071
**2**	26.0	0.098	22.7	0.012	7.5	0.090
**3**	34.0	15.4	53.6	0.008	12.1	0.062
**4**	21.0	1.0	34.5	5.1	6.4	0.009
**5**	3.2	9.5	4.1	0.007	2.5	0.004
**6**	19.0	8.3	38.0	0.002	1.8	0.89
**7**	31.0	0.73	8.2	0.01	4.9	0.023
**8**	nd	18.9	37.8	0.83	2.8	0.003
**9**	15.0	13.7	23.1	0.09	0.83	0.052
**10**	0.18	0.93	19.4	0.43	0.38	0.11
**11**	10.1	0.074	41.6	0.97	0.75	0.09
**12**	>100	0.007	9.1	0.08	2.7	0.10
**13**	56.0	0.006	11.7	0.021	2.0	0.011
**14**	89.0	0.005	35.2	0.037	>100	0.007
**15**	47.0	0.009	18.2	0.68	>100	0.081
**16**	>100	0.41	68.3	0.10	>100	0.34
**17**	>100	0.26	71.9	0.33	>100	0.076
**18**	11.5	0.004	57.3	0.09	>100	1.15
**19**	64.0	0.15	12.0	0.10	0.14	0.074

nd: not determined.

**Table 4:** In vitro fungal β-CAs activation data (K_A_) with amine derivatives and amino acids (**1**–**19**). Each value is the mean from three different determinations with errors in the range of 5–10%.

Compound	K_A_ (μM)					
	scCA	CaNce103	CgCA	Can2	MgCA	MreCA
**1**	82.0	24.1	37.0	45.0	29.3	12.8
**2**	85.0	19.5	21.2	47.2	18.1	1.8
**3**	86.0	15.5	24.1	44.1	34.1	3.0
**4**	86.0	8.4	15.7	45.2	10.7	0.76
**5**	91.0	19.2	22.8	28.7	10.1	0.32
**6**	90.0	43.0	12.1	42.1	12.5	0.89
**7**	85.0	46.1	9.5	29.5	15.7	4.1
**8**	84.0	nd	7.1	nd	25.1	7.8
**9**	90.0	0.96	23.3	43.3	8.31	0.87
**10**	89.0	2.5	15.1	35.1	13.7	0.70
**11**	21.3	23.7	31.6	30.4	13.4	0.61
**12**	20.4	18.4	27.4	33.2	10.9	0.90
**13**	15.0	28.6	16.7	46.7	14.2	0.82
**14**	13.1	18.5	27.6	34.6	9.43	2.7
**15**	0.95	13.2	10.8	32.8	0.72	0.015
**16**	16.2	29.1	15.0	47.0	6.1	0.34
**17**	11.2	30.2	16.3	46.3	7.3	2.1
**18**	9.3	17.3	14.9	44.9	0.81	0.25
**19**	10.2	25.4	10.1	40.1	5.8	0.33

nd: not determined.

**Table 5:** In vitro other β-CAs activation data (K_A_) with amine derivatives and amino acids (**1**–**19**). Each value is the mean from three different determinations with errors in the range of 5–10%.

Compound	K_A_ (µM)									
	Cab	mtCA3	VchCAβ	EcoCAβ	BsuCA1	FtuCA	LdcCA	EhiCA	TvaCA1	BpsCAβ
**1**	69.0	18.2	20.3	36.0	1.8	40.7	8.2	78.7	20.1	31.6
**2**	57.0	32.5	18.0	23.7	12.3	78.3	4.1	9.8	24.5	0.98
**3**	70.0	30.6	15.4	12.0	1.2	69.1	9.2	16.5	23.6	3.42
**4**	10.3	44.1	5.1	15.4	1.1	75.0	3.9	10.1	16.3	0.075
**5**	16.9	8.9	4.2	18.3	1.2	34.1	4.0	5.2	5.1	0.009
**6**	41.0	43.7	5.9	11.5	13.7	30.5	6.2	4.9	3.6	0.007
**7**	10.5	28.9	6.1	9.9	1.4	˃100	8.1	4.5	4.9	0.002
**8**	19.2	17.6	0.94	17.9	0.95	˃100	1.3	1.1	3.0	0.001
**9**	11.4	30.0	8.4	10.7	2.1	˃100	1.6	16.6	12.1	0.003
**10**	15.6	9.7	6.3	3.1	2.3	44.8	5.5	4.1	11.0	1.89
**11**	89.0	40.5	7.2	7.3	1.2	˃100	15.9	8.1	3.5	0.0009
**12**	76.0	34.2	9.5	18.5	3.7	˃100	0.74	7.4	8.4	0.012
**13**	62.0	10.3	1.4	2.8	4.3	˃100	0.62	4.9	9.1	0.006
**14**	51.0	12.1	1.2	11.3	1.5	˃100	0.81	30.8	12.6	0.027
**15**	11.5	52.2	8.7	9.1	0.70	˃100	4.9	25.6	8.3	0.016
**16**	18.7	43.3	0.18	48.7	1.6	46.3	0.23	˃100	9.5	0.94
**17**	40.0	45.9	1.0	17.2	5.2	˃100	0.012	˃100	12.0	0.004
**18**	13.8	50.3	0.24	14.1	43.1	51.8	0.009	43.8	11.8	0.073
**19**	18.5	52.0	12.8	17.4	9.6	˃100	0.94	˃100	14.5	0.002

**Table 6:** In vitro activation data (K_A_) of the remaining CA families with amine derivatives and amino acids (**1–19**). Each value is the mean from three different determinations with errors in the range of 5–10%.

Compound	K_A_ (µM)									
	γ-CAs						δ-CAs	ζ-CAs	η-CAs	ι-CAs
	Zn-Cam	Co-Cam	VchCAγ	BpsγCA	PhaCA	CpsCA	TweCAδ	Zn-TweCAζ	PfaCA	BteCAi
**1**	68.0	˃100	1.0	24.7	12.6	47.5	0.75	0.81	1.1	8.6
**2**	46.0	73.0	14.2	0.086	9.4	35.9	4.9	7.2	2.2	6.2
**3**	68.0	70.0	0.73	1.7	15.8	˃100	2.2	15.4	0.43	36.5
**4**	42.0	24.0	0.24	0.13	3.2	15.4	1.2	9.6	0.75	9.4
**5**	38.0	47.0	0.008	0.43	7.1	21.3	0.93	8.5	5.2	10.2
**6**	33.0	68.0	0.40	0.052	13.9	36.8	0.69	1.8	8.5	6.1
**7**	24.0	53.0	0.12	0.20	1.0	19.5	1.5	0.98	1.0	8.0
**8**	nd	nd	0.10	32.8	7.4	18.4	0.051	0.62	8.6	7.3
**9**	39.0	38.0	0.19	0.072	1.1	4.8	2.1	3.2	0.12	4.3
**10**	37.0	41.0	0.13	0.98	0.72	11.2	6.2	2.9	0.39	11.7
**11**	72.0	22.0	0.69	0.009	3.3	17.2	18.9	7.9	1.0	6.9
**12**	63.0	9.2	0.31	0.12	6.5	20.6	1.3	1.3	9.9	6.0
**13**	38.0	0.97	0.17	0.10	9.1	34.8	0.90	3.1	7.2	13.3
**14**	54.0	18.4	0.45	0.014	8.7	32.1	0.51	10.1	10.0	8.7
**15**	39.0	8.9	0.11	0.019	17.5	79.8	2.4	0.092	2.4	9.7
**16**	11.4	8.7	0.14	2.4	2.4	21.5	5.3	0.88	3.7	24.1
**17**	24.0	18.5	0.26	0.034	18.7	38.2	8.2	0.85	6.8	21.5
**18**	10.1	16.1	0.071	0.018	15.1	33.0	4.4	0.12	0.71	3.9
**19**	45.0	38.0	0.054	0.015	10.1	34.2	7.4	0.15	5.3	12.0

nd: not determined.

**Table 7:** In vitro activation data of selected CA families with six newly synthesized tripeptides (**76-81**). Each value is the mean from three different determinations with errors in the range of 5–10%.

Compound	NH_2_-Xaa_1_-Xaa_2_-Xaa_3_-NH_2_			K_A_ (µM)			
	Xaa_1_	Xaa_2_	Xaa_3_	VchCAβ	mtCA3	VchCAγ	BpsCAγ
**76**	Tyr	Phe	Asp	3.5	8.4	14.7	10.1
**77**	His	Phe	Glu	1.2	6.3	5.8	1.6
**78**	Glu	Ile	Thr	1.1	4.3	11.9	3.7
**79**	Gln	Asp	Ser	0.21	15.8	12.9	6.2
**80**	Asn	Asp	Ser	7.2	18.1	10.6	0.95
**81**	Glu	Phe	Glu	4.2	9.4	2.7	5.2

## Data Availability

Data are contained within the article.
